# Relationship Between Area Deprivation Index (ADI) Ranking and Targeted Multidrug Resistant Organisms (MDROs) in Philadelphia, 2018-2023

**DOI:** 10.1017/ash.2025.206

**Published:** 2025-09-24

**Authors:** Taseen Karim, Tiina Peritz, Jenna Scully, Jane Gould

**Affiliations:** 1 Philadelphia Department of Public Health; 2 NA

## Abstract

**Background:** Antimicrobial-resistant pathogens cause more than 2.8 million infections and 35,000 deaths annually in the U.S. Risks for antimicrobial-resistant infections are likely tied to social determinants of health. We assessed the relationship between ADI and targeted MDROs reported (mandatory and voluntary) to the Philadelphia Department of Public Health (PDPH), specifically: carbapenem-resistant Enterobacterales, Acinetobacter baumannii, Pseudomonas aeruginosa and Candida auris. **Method:** Confirmed MDRO case data were obtained from PDPH’s Communicable Disease Management System, PhilaVax, and CDC’s National Healthcare Safety Network for 04/2018 through 12/2023. MDRO patient home addresses were geocoded to census block groups, spatially mapped to zip codes using ArcGIS, and matched to ADI national percentile rankings and state decile rankings obtained from University of Wisconsin School of Medicine and Public Health’s Neighborhood Atlas website. Descriptive analysis using American Community Survey Data, calculation of MDRO prevalence rates by zip code, and Pearson correlation coefficients and simple linear regression between the MDRO cases and ADI were conducted using SAS 9.4. Missing and unknown values were excluded from descriptive analysis. **Result:** We analyzed 2436 MDRO cases reported to PDPH. Cases with race data (n=2138) were 51.9% Black and 34.1% White, compared to 43.3% Black and 38.0% White Philadelphia County population in 2023. Hispanic ethnicity was reported for 8.6% of cases (n=2101), while Hispanics represented 15.8% of the county population in 2023. Most patients with MDROs were ≥60 yo (60.1%). That age group was 55.5% male compared to the 42.5% male ≥60 yo county population. Most MDRO cases, 1846/2436 (75.8%), matched to Philadelphia zip codes and 1515/2436 (62.2%) matched to a census block ADI ranking. Three zip codes had a prevalence rate of >20 (M = 12, SD = 8; range 2-53) per 10,000 persons. Home address history for 356/2436 (14.6%) cases matched one or more congregate facilities, the majority of which matched a long-term care facility (286/2436, 11.7%). ADI percentiles and deciles positively correlated with the number of MDRO cases, r(86) = .62, p <.0001 and r(8) = .82, p = .0036, respectively. There was a significant effect of ADI percentiles and deciles on number of MDRO cases, F1,86 = 53.4, p **Conclusion:** There is a significant positive relationship between ADI ranking and MDROs in Philadelphia. The findings suggest a critical need for equitable outreach and interventions to address MDROs in both congregate facilities and communities experiencing greater deprivation.

